# The radiologist’s role in a multidisciplinary approach to cancer in pregnancy

**DOI:** 10.1007/s00261-023-03809-0

**Published:** 2023-01-27

**Authors:** Joelle Harwin, Priyanka Jha, Annalisa Post, Jordyn Silverstein, Katherine Van Loon, Liina Poder

**Affiliations:** 1 Department of Radiology and Biomedical Imaging, University of California, San Francisco, CA USA; 2 Department of Obstetrics, Gynecology and Reproductive Sciences, University of California, San Francisco, CA USA; 3 Department of Medicine, University of California, San Francisco, CA USA; 4 Department of Hematology/Oncology, Department of Medicine, University of California, San Francisco, CA USA

**Keywords:** Pregnancy-associated cancer, Malignancy in pregnancy, Multidisciplinary care, Radiology

## Abstract

Pregnancy-associated cancer (PAC) occurs in approximately 1 in 1000 pregnancies, and the incidence is expected to rise due to delayed childbearing (Silverstein et al. in JCO Oncol Pract 16:545–557, 2020; Woitek et al. in ESMO Open 1:e000017, 2016). Diagnosis and management of PACs are challenging and diagnosis is often delayed as symptoms may overlap with physiologic changes of pregnancy (Jha et al. in RadioGraphics 42:220005, 2022). These patients are best cared for by a multidisciplinary healthcare team composed of experts (Silverstein et al. in JCO Oncol Pract 16:545–557, 2020). Management of these patients must balance optimal maternal care with potentially harmful fetal effects. This involves honest, forthright, and sometimes difficult discussions between the care team and the patient throughout the entirety of care. Radiologists play a significant role in timely cancer diagnosis, staging and follow-up during and after pregnancy, accurate determination of gestational age, and in assessing fetal growth and well-being throughout pregnancy.

## Introduction

Maternal cancer occurs in approximately 1 in 1000 pregnancies [1]. As maternal age is increasing, the incidence of PAC is likely to increase [2]. Breast cancer, cervical cancer, Hodgkin's lymphoma, and melanoma are the most frequently diagnosed malignancies during pregnancy [3]. Oncologic management of the pregnant patient is complicated, as providing optimal maternal care must be balanced with minimizing harmful effects to the fetus. Due to this complexity of care, these patients should be managed by a collaborative multidisciplinary healthcare team. The radiologist is an important member of this care team in guiding imaging modality selection and providing radiation risk education. Because of the scope of practice, radiologists are often more familiar with the wide variety of oncologic processes and are integral in initial guidance of maternal fetal medicine specialists in referring the patient to an appropriate subspecialty care service.

## Presentation

Diagnosing maternal cancer during pregnancy is challenging and therefore often delayed, with over 50% of PACs diagnosed in the postpartum period [4]. A contributing factor may be that some symptoms associated with pregnancy overlap with symptoms of malignancy, such as anemia, breast tissue fluctuations, and fatigue [2]. Even physiologic serum lab values in the pregnant patient can overlap with abnormal values used to detect or trend malignancy. For example, elevated serum levels of cancer antigen-125 (CA-125) are often associated with ovarian cancer; however, this can be physiologically elevated in pregnant patients [4]. Diagnosis may also be delayed due to patient or provider hesitancy to thoroughly investigate suspicious symptoms during pregnancy out of concern for fetal harm from diagnosis.

**Fig. 1:**
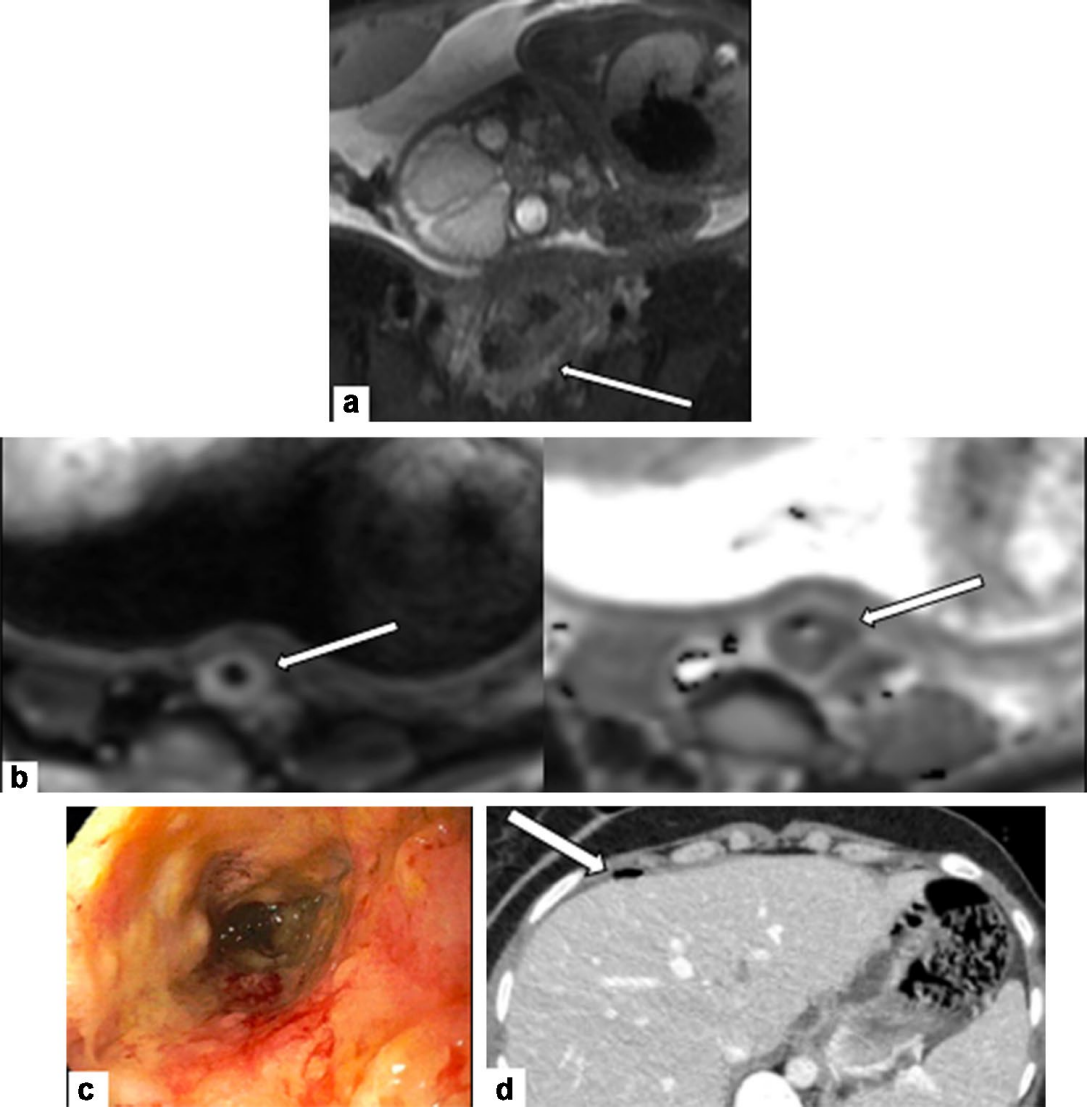
37-year-old pregnant patient, at 28 weeks of gestation, presenting with two months of recurrent diarrhea and rectal bleeding. Initial stool microscopy was positive for Giardia and flexible sigmoidoscopy noted mild proctitis. The patient’s symptoms persisted despite completion of treatment for Giardia and she was admitted for an expedited work-up of suspected colitis. **a** Axial T2-weighted fat-saturated MRI through the pelvis demonstrated circumferential, long segment thickening of the sigmoid colon wall (arrow), which was favored to represent colitis. **b** Retrospectively, diffusion-weighted MRI sequences demonstrated reduced diffusion of the colonic wall (arrow), which should have alerted the radiologist to possible underlying malignancy. Despite extensive treatment, the patient’s symptoms worsened and biopsy was recommended. **c** Repeat flexible sigmoidoscopy demonstrated erythematous, friable, and ulcerated sigmoid colonic tissue. Biopsies taken from this region were positive for adenocarcinoma. The patient was started on chemotherapy at 30 weeks of gestation. During her hospital course, the patient developed worsening, unexplained abdominal pain. After a detailed risks and benefits discussion with the patient, the shared decision was made to perform a CT abdomen/pelvis. **d** Axial contrast-enhanced CT abdomen/pelvis demonstrated new pneumoperitoneum (arrow) and **e** a rim-enhancing fluid and gas collection (arrowheads) consistent with sigmoid perforation. This necessitated urgent delivery via cesarean section at which time a concomitant left colonic resection was performed. Surgical pathology confirmed adenocarcinoma. During surgery, the patient was also found to have peritoneal disease which the team is planning to treat with systemic chemotherapy, peritoneal stripping, and hyperthermic intraperitoneal chemotherapy

Sometimes diagnosis of PAC is incidentally discovered during routine pregnancy evaluation. Since the American College of Medical Genetics and Genomics (ACMG) statement in 2013, use of noninvasive prenatal screening for fetal aneuploidies has become widespread [5]. This maternal serum screen analyzes circulating cell-free DNA(cfDNA) from placental cells [6]. In some malignancies, circulating cfDNA can also arise from cancer cells [7]. In this scenario, prenatal cfDNA platforms may product nonspecific abnormal results, though some platforms can specifically identify tumor cfDNA [7]. Nonspecific abnormal results should first prompt further evaluation with diagnostic genetic testing through amniocentesis or chorionic villus sampling to rule out fetal aneuploidy. Discordance between an abnormal cfDNA screen and normal fetal karyotype should raise a question of possible occult maternal malignancy and further investigation, including imaging, should be pursued [6]. One proposed algorithm for maternal evaluation after discordant cfDNA results includes history and physical exam, complete blood count, comprehensive metabolic panel, Papanicolaou test, fecal occult blood test, and a chest x-ray [8]. If unrevealing, a whole-body non-contrast MRI is suggested [8]. (Fig. 1) While this algorithm is a useful road map, a patient’s unique history and physical exam may indicate a different path. For example, in a patient with a family history of breast cancer, breast ultrasound and/or mammography might be best first steps.

It is important to note, however, that most maternal cancers are undetectable by cfDNA screening, so a normal cfDNA result does not exclude maternal malignancy [4].

Given the unique challenge that PACs are often discovered unexpectedly and diagnosis is delayed, prompt mobilization of a multidisciplinary care team is of paramount importance. This team should include, but does not need to be limited to, radiology, maternal fetal medicine, medical oncology, surgical oncology, and pathology. The patient should be promptly seen by a maternal–fetal medicine provider who will then collaborate with this group of experts to aid in diagnosis and generation of a treatment plan in a timely manner (Fig. 1). It is essential this multidisciplinary team meet in-person or virtually, with all members present. Once the case is reviewed, the treatment options must be discussed with the patient to consider their options. This process should remain patient centered, considering the patient’s values, resources, and cultural and religious beliefs.

## Imaging

Imaging is intimately associated with diagnosis, tissue sampling, staging, and surveillance of malignancies and the role of imaging in pregnancy-associated cancers is no exception. In pregnant patients, selecting the appropriate imaging modality is crucial as diagnostic ability must be weighed against potentially harmful maternal and fetal effects. Radiologists should provide thorough counseling on the safety of imaging and image-guided procedures during pregnancy.

**Fig. 2:**
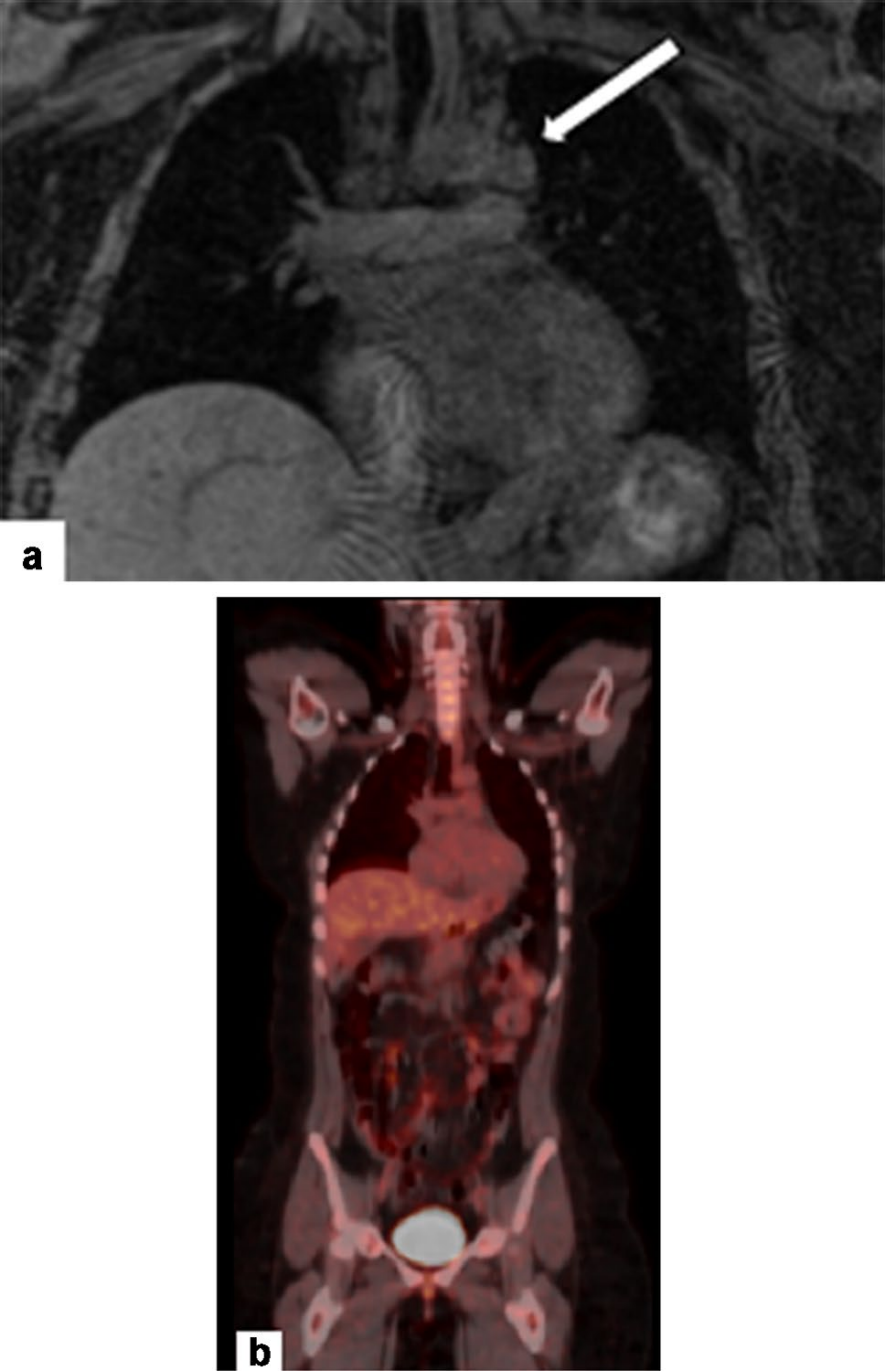
25-year-old pregnant patient in early first trimester, presenting with a palpable left supraclavicular mass. This was evaluated by ultrasound (not shown) which revealed an enlarged and abnormal lymph node corresponding to the patient’s palpable abnormality. Subsequent evaluation with fine needle aspiration and core biopsy, under ultrasound guidance, revealed Hodgkin’s lymphoma, at which time the patient was 8 weeks pregnant. Due to concern for fetal safety, staging was performed with a whole-body non-contrast MRI, instead of a PET-CT. **a** Coronal T1-weighted fat-saturated MRI demonstrated supraclavicular and mediastinal adenopathy (arrow). Chemotherapy was deferred until the second trimester. Maternal fetal medicine deemed no indication for early delivery based on her malignancy diagnosis. **b** Coronal fused images from the follow-up PET-CT after term delivery showed no evidence of disease, compatible with remission

Ultrasound (US) and magnetic resonance imaging (MRI) do not expose patients to ionizing radiation and are therefore favorable imaging modalities in the pregnant patient (Fig. 2). There are no limitations to the use of non-contrast MRI in pregnancy; however, the MRI contrast agent gadolinium is controversial. Gadolinium crosses the placenta and enters the fetal circulation and amniotic fluid where is it recirculated and can be ingested by the fetus. The largest retrospective study on gadolinium use in pregnancy found an increased risk of rheumatologic, inflammatory, or infiltrative skin conditions in offspring [9]. There are conflicting data on the increased risk of stillbirth and neonatal death in a fetus exposed to gadolinium in utero and longitudinal, prospective studies will be helpful to address this concern [10]. Based on the limited data, gadolinium use in pregnancy is limited to situations in which the benefits to maternal or fetal outcomes significantly outweigh associated risks [11]. Many institutions perform gadolinium-enhanced exams in pregnancy with documented informed consent [11] (Fig. 2).

Imaging modalities which expose pregnant patients to ionizing radiation include radiographs, computed tomography (CT), nuclear imaging, and positron emission tomography. Ionizing radiation risk to the fetus depends on gestational age at the time of exposure and the absorbed fetal dose. The absorbed fetal dose varies based on a variety of factors, including which maternal body part is imaged and if the gravid uterus is included in the field of view [12]. A single diagnostic CT usually exposes a fetus to an absorbed dose of well under 50 mGy. Below this threshold, there is no risk of fetal lethality, intellectual impairment, teratogenicity, growth impairment, or sterility [13]. However, a single diagnostic imaging study with high radiation dose and including the pelvis can expose the fetus to an absorbed dose of 10 mGy or greater, which is associated with an increased risk of childhood leukemia [12]. Although the absolute risk is increased from approximately 1/3000 to 1/5000 live births, these imaging studies may be considered when critical for decision-making [11].

Radiation-based imaging selection should adhere to the “as low as reasonably achievable” (ALARA) principal [13]. If the decision is made to pursue ionizing radiation studies, informed consent outlining the risks and benefits, and an estimate of the absorbed maternal and fetal radiation dose, should be provided [4, 12]. In addition, radiation doses are potentially additive and therefore accurate estimation of the absorbed dose over multiple examinations may warrant consultation with a radiation physicist [13].

## Management

Radiologists also assist in discerning which lesions are amenable to biopsy and often perform image-guided biopsies for tissue diagnosis, assessment of tissue genetics, and staging. Once diagnosis and staging have occurred, a comprehensive discussion with the patient about the diagnosis, prognosis, treatment options, potential harmful effects to the fetus, and consequences of deferring treatment during pregnancy should occur. In certain scenarios, a discussion about discontinuing the pregnancy may be appropriate [4]. Given the US Supreme Court’s recent decision to overturn *Roe v. Wade*, the care team must be aware of state-specific legislation regarding pregnancy termination in order to best counsel the patient regarding logistics of their reproductive options [14]. The repeal of *Roe v. Wade* has tremendous consequences for both clinicians and patients, the far-reaching effects of which are beyond the scope of this article. The American Society for Reproductive Medicine recommends patients who desire fertility preservation be offered consultation with a reproductive endocrinologist before beginning treatment to discuss anticipated impact on fertility and options for oocyte retrieval and preservation [15].

Radiologic confirmation of gestational age is critical to inform therapeutic decision-making including the safety of systemic therapies and surgical timing [4]. When chemotherapeutics are considered during pregnancy, patients should be counseled by oncologic and obstetric providers who are well versed in fetal effects specific to each drug class [16]. Chemotherapy administered within the first 2 weeks after conception can interrupt implantation, resulting in a miscarriage. However, if the embryo survives, it is often expected to develop normally [17]. Organogenesis occurs during the first 2–10 weeks of gestation. Administration of chemotherapy should be avoided during this time as it is associated with increased risks of congenital malformations [18]. Some chemotherapies can be safely administered during the second and third trimesters, without an increased risk of congenital anomalies [18]. Patients should be informed that the primary risk of chemotherapy administration during pregnancy is preterm birth and neonates being born small for gestational age (SGA), with the attendant complications of prematurity and SGA [18–20].

**Fig. 3:**
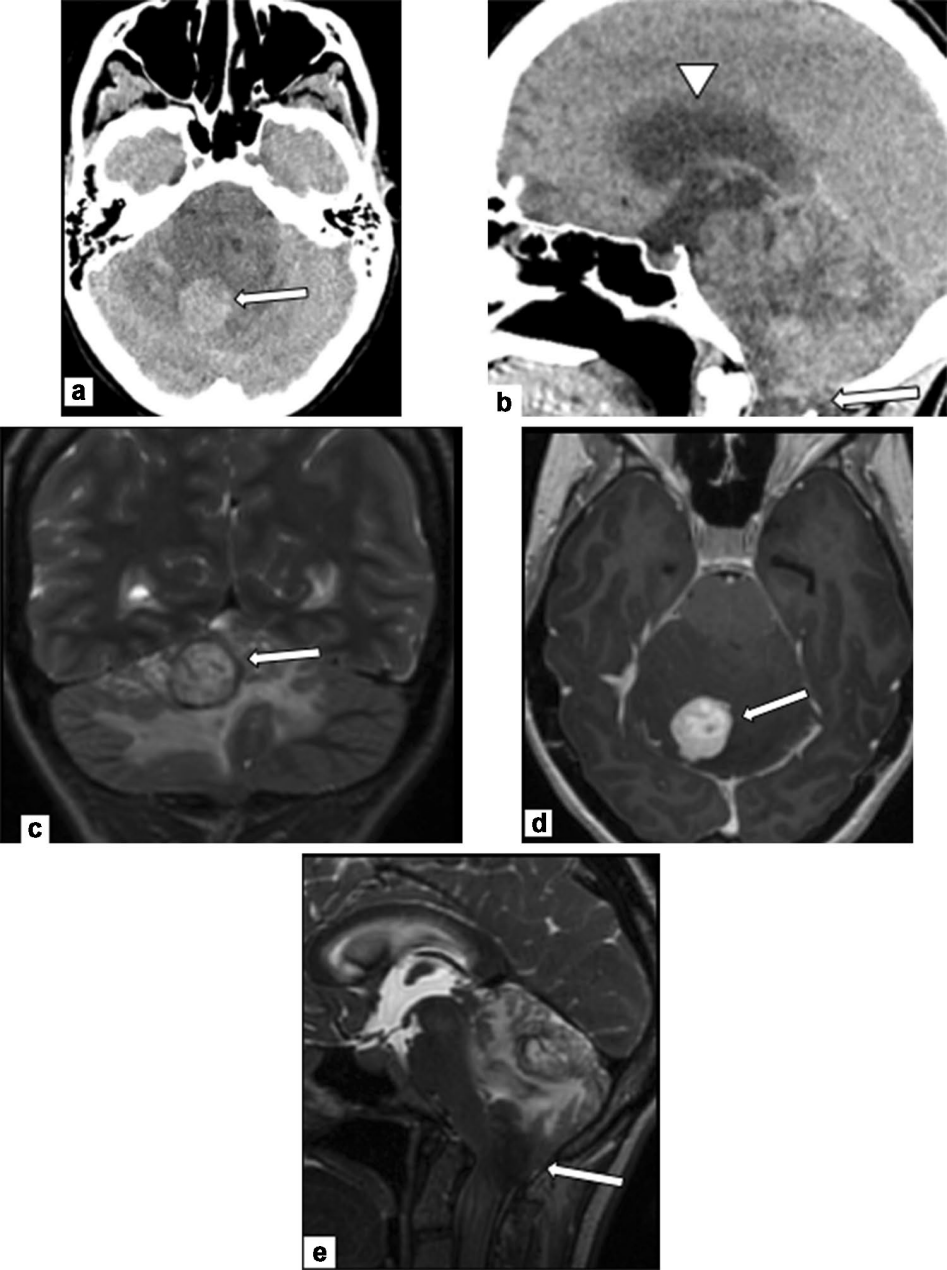
30-year-old pregnant patient, at 32 weeks of gestation, presenting with persistent headaches and nausea. **a** Axial non-contrast CT demonstrated a dense mass centered in the right cerebellum with surrounding edema (arrow). **b** Sagittal non-contrast CT demonstrated downward cerebellar tonsillar herniation (arrow) and acute obstructive hydrocephalous (arrowhead). Emergent treatment was initiated to reduce intracranial pressure. Once the patient was stable, a multidisciplinary team discussed next best steps in management. Despite theoretical fetal risks, the decision was made to pursue an MRI with contrast. **c** Coronal T2-weighted MRI images demonstrated a hyperintense mass (arrow) with a peripheral rim of hemosiderin and vascular flow voids. **d** T1-weighted contrast-enhanced MRI axial images demonstrated a well-circumscribed, enhancing mass, (arrow) most compatible with a hemangioblastoma. **e** Sagittal T2-weighted MRI images demonstrated continued marked mass effect with tonsillar herniation (arrow), upper cervical cord edema, and papilledema. The patient was scheduled for urgent surgery. However, the day before her surgery, she became preeclamptic and was delivered by emergency cesarean section at 33 weeks. After delivery, the patient underwent craniotomy for tumor resection with pathology confirming hemangioblastoma. After resection, the patient reported resolution of her presenting symptoms and is being followed with serial imaging

Surgical intervention can be performed at centers with appropriate expertise, if urgently indicated. Retrospective studies of nonobstetric surgery during pregnancy indicate either no change, or a small increase in the risk of complications such as miscarriage, low birth weight, and premature delivery [21]. Any urgently indicated procedure, such as definitive surgery for cancer that will improve maternal prognosis, should be offered without delay by experienced providers (Fig. 3). Clinicians should be familiar with state-specific reproductive health legislation, as certain state mandates may be problematic for providers who offer procedures that benefit the mother but compromise the fetus. There are several alterations to surgical approach to maximize maternal and fetal safety including left lateral positioning after 20 weeks, consideration of fetal monitoring after viability, care from an anesthesiologist with expertise in pregnancy physiology, and consideration of perioperative anticoagulation [22]. When possible, it is preferable to perform pelvic or abdominal surgery in the early second trimester before the gravid uterus interferes with surgical access [4]. In some cases, surgical tumor resection can coincide with cesarean section (Fig. 3).

Radiation therapy is seldom used during pregnancy due to the abovementioned risks related to fetal dose of ionizing radiation and because it is often an adjuvant treatment that can be deferred until postpartum. In rare cases, however, radiation therapy (not including the pelvis) can be considered during pregnancy [23]. In such instances, consultation with a radiation physicist should be performed to guide treatment.

These patients with PAC should be cared for by maternal–fetal medicine throughout their pregnancy, with close fetal surveillance [19]. Monthly fetal growth assessments and antenatal testing initiation by 32 weeks are typically recommended [4]. Timing of delivery is dependent on many factors including whether treatments are being withheld until after delivery, avoidance of chemotherapy hematologic suppression nadirs, and fetal growth status. After birth, the placenta should be evaluated by pathology for metastasis [4].

## Conclusion

Though PAC is relatively rare, the incidence is expected to rise [2]. Evaluation and management of PACs are complex and these cases are best served by a multidisciplinary healthcare team composed of experts [19]. This team should provide forthright patient-centered counseling throughout the entirety of care. Radiologists play a significant role in timely cancer diagnosis, accurate determination of gestational age, work-up of occult cancer detected incidentally, diagnosis and staging, radiation safety, and monitoring fetal well-being and growth throughout the pregnancy.
